# Genetic analysis of the relation between *IL2RA*/*IL2RB* and rheumatoid arthritis risk

**DOI:** 10.1002/mgg3.754

**Published:** 2019-05-27

**Authors:** Yonghui Yang, Shan Yuan, Meihua Che, Haiyin Jing, Limin Yuan, Kuaini Dong, Tianbo Jin

**Affiliations:** ^1^ Clinical Laboratory Xi'an 630 Hospital Yanliang, Xi'an Shaanxi; ^2^ Key Laboratory of Molecular Mechanism and Intervention Research for Plateau Diseases of Tibet Autonomous Region School of Medicine, Xizang Minzu University Xianyang Shaanxi China; ^3^ Key Laboratory of High Altitude Environment and Genes Related to Diseases of Tibet Autonomous Region School of Medicine, Xizang Minzu University Xianyang Shaanxi China; ^4^ Key Laboratory for Basic Life Science Research of Tibet Autonomous Region School of Medicine, Xizang Minzu University Xianyang Shaanxi China

**Keywords:** case–control study, genetic polymorphism, *IL2RA*, *IL2RB*, rheumatoid arthritis (RA)

## Abstract

**Background:**

The biological mechanisms driving disease chronicity in rheumatoid arthritis (RA) are largely unidentified. Therefore, we aimed to determine genetic risk factors for RA.

**Methods:**

In this case–control study, which includes samples from 499 patients and 507 healthy controls, six single‐nucleotide polymorphisms (SNPs) in interleukin‐2 receptor subunit alpha (*IL2RA*) and *IL2RB* were selected. Genotyping was performed using the Agena MassARRAY platform, and the statistical analyses were performed using the chi‐squared and Fisher's exact tests, genetic model analysis, and haplotype analysis.

**Result:**

In the allele model, using the chi‐squared test, the result showed that rs791588 in *IL2RA* was associated with a decreased RA risk (odds ratios [OR] = 0.74, 95% confidence intervals [CI] = 0.62–0.89, *p* = 0.0014) after adjusting for age and gender. In the genetic model, logistic regression analyses revealed that rs791588 was associated with a decreased risk of RA under the codominant model, dominant model, recessive model, and log‐additive model. Stratification analysis revealed that two SNPs (rs791588 and rs2281089) were significantly associated with a reduced RA risk in an allele and genetic model after stratification by gender or age (*p* < 0.05). In addition, the haplotypes “C_rs12569923_G_rs791588_” and “C_rs12569923_T_rs791588_” of *IL2RA* was associated with an increased risk of RA adjusted by age and gender (OR = 1.35, 95% CI: 1.12–1.64, *p* = 0.0016; OR = 1.24, 95% CI: 1.03–1.48, *p* = 0.021).

**Conclusion:**

This finding indicates that the inherited altered genetic constitution at *IL2RA* may predispose to a less destructive course of RA.

## 1 INTRODUCTION

Rheumatoid arthritis (RA), one of the most common systemic autoimmune disorders, is characterized by peripheral synovial joint inflammation, which ultimately leads to long‐term joint damage, loss of function, a poor quality of life, and increases mortality (Silman & Pearson, 2002). A thorough comprehension of the mechanisms promoting disease persistence is required to derive targeted interventions aiming to reduce the chronic nature of RA. Although the exact causes of RA remain unknown, immunological dysregulation by inflammatory cytokines has been shown to be involved in driving the inflammation and synovial cell proliferation that characterize joint destruction in RA patients (Pawlik et al., 2016; Pei et al., 2013).

Advances in genotyping technology and the use of single‐nucleotide polymorphism (SNP) assays have facilitated the application of whole genome association approaches to link genetic variants with disease susceptibility (Bowes & Barton, 2008; Criswell et al., 2006). Heritability studies have revealed a critical role of genetic susceptibility in the progression of joint destruction in RA; the heritability is estimated as 45%–58% (Adeline et al., 2014). Several SNPs were identified to be associated with RA predisposition. Large genome‐wide association studies (GWAS) have identified more than 30 loci involved in RA pathogenesis (De, 2011). Genetic factors including human leukocyte antigen gene (*HLA*) (Nepom et al., 2010), interleukin genes (*IL23R*, *IL6*, *IL17*, and *IL12B*) (Chabaud, Fossiez, Taupin, & Miossec, 1998; Chang et al., 2010), autoimmune regulator gene (*AIRE*) (José‐Raúl et al., 2013), protein tyrosine phosphatase 22 (*PTPN22*) gene (Hinks et al., 2010), and solute carrier family 22 member 4 (*SLC22A4*) (Newman et al., 2010) have been implicated in the pathogenesis of RA.

Interleukin 2 (*IL‐2*) was initially identified as an autocrine product from activated T cells. It was found that *IL‐2* plays a crucial role in the maintenance of system homeostasis and self‐tolerance (Churlaud et al., 2014; Shows et al., 1984). *IL‐2* receptor alpha (*IL2RA*, OMIM: 147730) and *IL‐2* receptor beta (*IL2RB*, OMIM: 146710) genes (located in 10p15 and 22q13, respectively) were reported to be associated with the development of autoimmune diseases and inflammatory diseases, such as type 1 diabetes (TID) (Ferjani et al., 2016), multiple sclerosis (Cavanillas et al., 2010), inflammatory bowel disease (Bouzid et al., 2013), and intermediate uveitis (Ewald, Martin, Navid, Wilfried, & Yosuf, 2015). However, little studies have investigated the association between genetic variants in *IL2RA* and *IL2RB* and the risk of RA. Therefore, we performed a case–control study to analyze the associations between *IL2RA* and *IL2RB* and the risk of RA.

## 2 MATERIALS AND METHODS

### 2.1 Subject recruitment and ethics committee statement

In total, 499 primary arthritis patients and 507 controls were enrolled in this study; all of whom were genetically unrelated Han Chinese. All participants were recruited from the Xi'an 630 Hospital. All primary arthritis patients were diagnosed with RA who were in clinical follow‐up of the rheumatology service for over 12 months. The control group was age‐ and gender‐matched healthy subjects without any inflammatory bone and joint disease or other diseases.

All participants were informed both in writing and verbally the procedures and purpose of the study, and they signed informed consent documents. The use of human tissue and the protocol in this study were strictly conformed to the principles expressed in the Declaration of Helsinki, and this study was carried out with approval from the ethics committee of the Xi'an 630 Hospital. All the subsequent research analyses were carried out in accordance with the approved guidelines and regulations.

### 2.2 SNP selection and genotyping

A GoldMag‐Mini Purification Kit (GoldMag Co. Ltd. Xian city, China) was used to extract genomic DNA from whole‐blood samples. DNA samples were stored at −20°C prior to analysis. At the same time, the concentrations and purity of the DNA were measured by using the NanoDrop 2000 (Thermo Fisher Scientific, Waltham, MA) at a wavelength of A260 and A280 nm.

Six tag SNPs (rs12569923, rs791588, rs12722498, rs2281089, rs3218264, and rs1573673) in *IL2RA* and *IL2RB* were selected for our study. These SNPs had minor allele frequencies (MAF) greater than 5% according to the 1000 Genomes Project (http://www.internationalgenome.org/). The selected SNPs were reported to be associated with inflammatory disease. The primers were designed online (https://agenacx.com/online-tools/). The PCR primers for each SNP are shown in Table S1. Agena MassARRAY Assay Design 4.0 software was used to design a multiplexed SNP MassEXTEND assay, and SNP genotyping was performed using the Agena MassARRAY RS1000 with manufacturer protocols. Agena Typer 4.0 software was used to perform data management and analyses.

### 2.3 Statistical analysis

All statistical analyses were performed using SPSS 19.0 software for Windows (SPSS, Chicago, IL). Genotyping results were performed by Agena Bioscience TYPER version software 4.0. Pearson’s chi‐squared test and independent sample Student's *t *test were applied to assess the differences in the distribution of demographic characteristics between cases and controls. Fisher's exact tests for Hardy–Weinberg equilibrium (HWE) were performed by comparing the observed and expected genotype frequencies to calculate the genotype frequencies among the controls. Odds ratios (OR) and 95% confidence intervals (CI) were calculated to estimate the association between the genes (*IL2RA* and *IL2RB*) and the risk of RA using unconditional logistic regression analysis after adjusting for age and gender (Bland & Altman, 2000). Four genotype model analyses (codominant, dominant, recessive, and log‐additive) were applied using PLINK software (Version 1.07) to evaluate the association between SNPs and the risk of RA. Haplotype construction and genetic association between polymorphism loci were assessed using the Haploview software package (version 4.2) and the SHEsis software (http://analysis.bio-x.cn/myAnalysis.php) (Barrett, Fry, Maller, & Daly, 2005; YongYongSHI & LinHE, 2005). All *p* values of statistical tests were two‐sided, and *p* < 0.05 was considered as statistically significant.

## 3 RESULTS

### 3.1 Characteristics of the participants

This study involved 1,006 subjects, including 499 patients (135 males and 364 females; age at diagnosis: 56.32 ± 10.02 years) and 507 healthy subjects (135 males and 372 females; age: 56.30 ± 12.68 years). The cases and controls were matched by age and sex, and there were no significant differences in the distributions of age and sex between RA patients and healthy controls (*p*﹥0.05) (Table 1).

**Table 1:** Characteristics of case and control participants

Variables	Case	%	Control	%	*p*
Total	499		507		
Gender					0.879^a^
Male	135	27.0	135	27.0	
Female	364	73.0	372	73.0	
Age (year, *SD*)	53.32 ± 10.02		56.30 ± 12.68		0.508^b^
﹤54	278	56.0	246	49.0	
﹥54	221	44.0	261	51.0	

a *p* values were calculated from two‐sided chi‐squared tests.

b *p* values were calculated by Student's *t* tests;

### 3.2 Associations between *IL2RA* and *IL2RB* SNPs and RA risk

Six SNPs in *IL2RA* and *IL2RB* were selected. Position, alleles, and minor allele frequency of these two SNPs were showed in Table 2. All SNPs were consistent with HWE (*p*﹥0.05). Pearson’s chi‐squared test was used to assess the association between SNP variants and the risk of RA. The frequency of the minor allele “G” of rs791588 was significantly lower in RA cases than in controls (32.7% vs. 39.6%), which suggested that “G” allele of rs791588 plays a protective role against RA risk (OR = 0.74, 95% CI = 0.62 – 0.89, *p* = 0.0014).

**Table 2:** Basic information of candidate SNPs and minor allele frequency between cases and control participants

SNP rs#	Chromosome	Alleles A/B	Gene(s)	MAF		*p*_‐HWE_	OR (95% CI)	*p* ^a^	*p^b^*
				Case	Control				
rs12569923	10	C/G	*IL2RA*	0.207	0.193	1.000	1.11 (0.89–1.38)	0.347	0.057
rs791588	10	G/T	*IL2RA*	0.327	0.396	0.925	0.74(0.62–0.89)	0.0014*	0.00023*
rs12722498	10	C/T	*IL2RA*	0.110	0.111	0.649	0.99(0.75–1.31)	0.963	0.1605
rs2281089	22	A/G	*IL2RB*	0.233	0.270	0.909	0.82(0.66–1.00)	0.053	0.0088
rs3218264	22	C/T	*IL2RB*	0.507	0.494	0.531	1.05(0.88–1.25)	0.562	0.0936
rs1573673	22	C/T	*IL2RB*	0.352	0.363	0.100	0.95(0.79–1.14)	0.594	0.099

Abbreviations: Alleles A/B, Minor/major alleles; CI, confidence interval; HWE, Hardy–Weinberg equilibrium; MAF, minor allele frequency; OR, odds ratio; SNP, single‐nucleotide polymorphism.

*p* ^a^ values were calculated using two‐sided chi‐squared test (the major allele of each SNP was a reference allele);

*p* ^b^ values were adjusted by Bonferroni correction.

* *p* ^a ^˂ 0.05 indicates statistical significance.

* *p* ^b^ *˂* 0.008 indicates statistical significance.

After adjusting for age and gender, four genotype models of *IL2RA* and *IL2RB* polymorphisms are shown in Table 3. There was a significant association between one SNP and the risk of RA. The “G/G” genotype of rs791588 in *IL2RA* was associated with a decreased risk of RA as revealed by the codominant model when compared to the “T/T” genotype (OR = 0.45, 95% CI = 0.29 – 0.69, *p* = 0.001). The “G/T‐G/G” genotype of rs791588 in *IL2RA* plays a protective role to reduce RA risk as revealed by the dominant model when compared to the “T/T” genotype (OR = 0.77, 95% CI = 0.60 –0.99, *p* = 0.046). The “G/G” genotype of rs791588 was related to a 0.48‐fold decreased risk of RA as revealed by the recessive model (OR = 0.48, 95% CI = 0.32–0.72, *p* = 0.00034) and 0.73‐fold decreased risk of RA as revealed by the log‐additive model (OR = 0.73, 95% CI = 0.61–0.88, *p* = 0.0012).

**Table 3:** Association between candidate SNPs and the risk of RA under genotype models

SNP	Model	Genotype	Genotype frequency		OR (95% CI)	*p*‐value
			Case	Control		
*IL2RA*						
rs12569923	Codominant	G/G	327	316	1	0.314
		C/G	154	172	1.15 (0.88–1.50)	
		C/C	18	19	1.07 (0.55–2.09)	
	Dominant	G/G	327	316	1	0.320
		G/C‐C/C	172	191	1.14 (0.88 –1.48)	
	Recessive	G/G‐G/C	481	488)	1	0.938
		C/C	18	19	1.03 (0.53–1.98)	
	Log‐additive	—	—	—	1.11 (0.88–1.38)	0.379
rs791588	Codominant	T/T	217	183	1	0.001*
		G/T	248	237	0.88 (0.67–1.15)	
		G/G	42	79	0.45 (0.29–0.69)	
	Dominant	T/T	217	183	1	0.046*
		G/T‐G/G	290	316	0.77 (0.60–0.99)	
	Recessive	T/T‐G/T	465	420	1	0.0003*
		G/G	42	79	0.48 (0.32–0.72)	
	Log‐additive	—	—	—	0.73 (0.61–0.88)	0.001*
rs12722498	Codominant	T/T	398	392	1	0.392
		C/T	106	96	1.09 (0.80–1.48)	
		C/C	3	7	0.43 (0.11–1.66)	
	Dominant	T/T	398	392	1	0.776
		T/C‐C/C	109	103	1.04 (0.77–1.42)	
	Recessive	T/T‐T/C	504	488	1	0.209
		C/C	3	7	0.42 (0.11–1.63)	
	Log‐additive	—			0.99 (0.75–1.32)	0.977
*IL2RB*						
rs2281089	Codominant	G/G	300	266	1	0.149
		G/A	178	195	0.81 (0.62–1.05)	
		A/A	29	37	0.69 (0.41–1.15)	
	Dominant	G/G	300	266	1	0.062
		A/G‐A/A	207	232	0.79 (0.61–1.01)	
	Recessive	G/G‐A/G	478	461	1	0.269
		A/A	29	37	0.75 (0.45–1.25)	
	Log‐additive	—	—	—	0.81 (0.67–1.00)	0.052
rs3218264	Codominant	T/T	116	124	1	0.781
		C/T	268	257	1.11 (0.82–1.51)	
		C/C	123	118	1.11 (0.77–1.58)	
	Dominant	T/T	116	124	1	0.482
		T/C‐C/C	391	375	1.11 (0.83–1.48)	
	Recessive	T/T‐T/C	384	381	1	0.846
		C/C	123	124	1.03 (0.77–1.38)	
	Log‐additive	—	—	—	1.05 (0.88–1.26)	0.581
rs1573673	Codominant	T/T	209	193	1	0.678
		C/T	239	248	0.89 (0.68–1.16)	
		C/C	59	57	0.96 (0.63–1.45)	
	Dominant	T/T	209	193	1	0.423
		T/C‐C/C	298	305	0.90 (0.70–1.16)	
	Recessive	T/T‐T/C	448	421	1	0.908
		C/C	59	57	1.02 (0.69–1.51)	
	Log‐additive	—	—	—	0.94 (0.78–1.15)	0.589

Abbreviations: AIC, Akaike's information criterion; BIC, Bayesian information criterion; CI, confidence interval; OR, odds ratios, RA, rheumatoid arthritis; SNP, single‐nucleotide polymorphism.

*p* values were calculated from Wald's test adjusted for age and sex.

* *p* ≤ 0.05 indicates statistical significance.

### 3.3 Stratification analysis

As shown in Table 4, we implemented a stratification analysis by gender and age to evaluate sex‐ and age‐specific associations between SNP alleles and RA risk. In the allele model, we discovered that rs791588 (*IL2RA*) and rs2281089 (*IL2RB*) significantly decreased the risk of RA in females (rs791588: OR = 0.49, 95% CI = 0.30–0.82, *p* = 0.006) and people aged under 54 (rs2281089: OR = 0.37, 95% CI = 0.21–0.68, *p* = 0.001 and OR = 0.72, 95% CI = 0.56–0.93, *p* = 0.011). In addition, rs791588 was associated with a decreased risk of RA in males (OR = 0.46, 95% CI = 0.27–0.78; OR = 0.35, 95% CI = 0.16–0.77, *p* = 0.0035; OR = 0.54, 95% CI = 0.38–0.78, *p* = 0.001) and the population over 64 years of age (OR = 0.43, 95% CI = 0.23–0.81, *p* = 0.009).

**Table 4:** Association between sex and age stratification and RA risk in allele and genotype models

SNP	Alleles	Male		Female		Age ≤ 54		Age﹥54	
		OR(95% CI)	*p^a^*	OR(95%CI)	*p^a^*	OR(95% CI)	*p^b^*	OR(95% CI)	*p^b^*
*IL2RA*									
rs12569923	G/G	1	0.353	1	0.739	1	0.675	1	0.189
	G/C	1.45 (0.86–2.47)		1.05 (0.77–1.44)		1.08 (0.74–1.59)		1.29 (0.88–1.90)	
	C/C	0.89 (0.23–3.44)		1.14 (0.53–2.45)		1.23 (0.46–3.30)		1.11 (0.44–2.76)	
	G	1	0.321	1	0.616	1	0.983	1	0.247
	C	1.25 (0.81–1.92)		1.07 (0.13–0.83)		0.99 (0.73–1.36)		1.20 (0.88–1.64)	
rs791588	T/T	1	0.003*	1	0.006*	1	0.001*	1	0.009*
	G/T	0.46 (0.27–0.78)		1.11 (0.81–1.51)		0.76 (0.52–1.11)		0.90 (0.61–1.33)	
	G/G	0.35 (0.16–0.77)		0.49 (0.30–0.82)		0.37 (0.21–0.68)		0.43 (0.23–0.81)	
	T	1	0.001*	1	0.085	1	0.011*	1	0.056
	G	0.54 (0.38–0.78)		0.83 (0.67–0.13)		0.72 (0.56–0.93)		0.77 (0.59–1.01)	
rs12722498	T/T	1	0.186	1	0.766	1	0.598	1	0.897
	T/C	1.52 (0.82–2.82)		0.97 (0.67–1.39)		1.12 (0.73–1.72)		1.03 (0.65–1.64)	
	C/C	—		0.58 (0.14–2.48)		0.95 (0.21–4.33)		—	
	T	1	0.495	1	0.681	1	0.703	1	0.673
	C	1.21 (0.69–2.13)		0.93 (0.67–1.28)		1.09 (0.74–1.58)		0.91 (0.60–1.39)	
*IL2RB*									
rs2281089	G/G	1	0.210	1	0.047*	1	0.004*	1	0.784
	G/A	1.06 (0.63–1.77)		0.73 (0.54–0.99)		0.54 (0.37–0.79)		1.16 (0.79–1.71)	
	A/A	0.38 (0.11–1.28)		0.78 (0.44–1.40)		0.57 (0.29–1.15)		1.09 (0.48–2.52)	
	G	1	0.377	1	0.087	1	0.001	1	0.406
	A	0.83 (0.55–1.25)		0.81 (0.65–1.03)		0.62 (0.46–0.82)		1.14 (0.85–1.54)	
rs3218264	T/T	1	1.239	1	0.782	1	0.844	1	1.049
	T/C	1.45 (0.80–2.64)		0.95 (0.67–1.35)		1.04 (0.68–1.59)		1.08 (0.70–1.66)	
	C/C	1.24 (0.60–2.59)		0.94 (0.62–1.43)		0.89 (0.53–1.51)		0.85 (0.51–1.42)	
	T	1	0.547	1		1	0.876	1	0.476
	C	1.11 (0.79–1.55)		0.97 (0.79–1.19)		0.98 (0.77–1.25)		0.90 (0.70–1.17)	
rs1573673	T/T	1	0.173	1	0.666	1	0.134	1	1.148
	T/C	0.73 (0.43–1.23)		0.95 (0.70–1.29)		0.75 (0.52–1.09)		1.01 (0.67–1.49)	
	C/C	1.06 (0.51–2.22)		0.89 (0.54–1.48)		1.15 (0.63–2.07)		0.74 (0.40–1.36)	
	T	1	0.791	1	0.662	1	0.980	1	0.351
	C	0.95 (0.67–1.35)		0.95 (0.77–1.18)		0.99 (0.77–1.29)		0.88 (0.68–1.14)	

Abbreviations: 95% CI, 95% confidence interval; OR, odds ratio; RA, rheumatoid arthritis; SNP, single‐nucleotide polymorphism.

*p^a^*‐values were calculated from Wald's test adjusted for age.

*p^b^*‐values were calculated from Wald's test adjusted for gender.

* *p* < 0.05 indicates statistical significance.

After stratification by age and gender in the genetic model (Table 5), rs791588 was significantly associated with a decreased risk of RA in males (dominant model: OR = 0.44, 95% CI = 0.27–0.72, *p* = 0.001 for the “G/T‐G/G” genotype; log‐additive model: OR = 0.55, 95% CI = 0.38–0.79, *p* = 0.001) and females (recessive model: OR = 0.47, 95% CI = 0.29–0.75, *p* = 0.002). Similarly, rs791588 plays a protective role to decrease RA risk in the population under 54 years of age (dominant model: OR = 0.66, 95% CI = 0.46–0.95, *p* = 0.024 for the “G/T‐G/G” genotype; log‐additive model: OR = 0.65, 95% CI = 0.50–0.85, *p* = 0.002) and over 54 years of age (log‐additive model: OR = 0.73, 95% CI = 0.55–0.96, *p* = 0.027).

**Table 5:** Association between sex and age stratification and RA risk in genetic models

Model	Genotype	Male		Female		Age ≤ 54		Age﹥54	
		OR (95% CI)	*p^a^*	OR (95% CI)	*p^a^*	OR (95% CI)	*p^b^*	OR (95% CI)	*p^b^*
rs12569923 (*IL2RA*）									
Dominant	G/G	1	0.206	1	0.684	1	0.624	1	0.203
	G/C‐C/C	1.39 (0.83–2.31)		1.06 (0.79–1.44)		1.10 (0.76–1.59)		1.27 (0.88–1.85)	
Recessive	G/G‐G/C	1	0.726	1	0.775	1	0.712	1	0.987
	C/C	0.78 (0.21–3.00)		1.12 (0.52–2.38)		1.20 (0.15–3.19)		1.01 (0.41–2.49)	
Log‐additive	—	1.25 (0.80–1.94)	0.328	1.06 (0.82–1.37)	0.656	1.09 (0.78–1.51)	0.586	1.19 (0.87–1.64)	0.276
rs791588 (*IL2RA*）									
Dominant	T/T	1	0.001*	1	0.774	1	0.024*	1	0.100
	G/T‐G/G	0.44 (0.27–0.72)		0.95 (0.71–1.29)		0.66 (0.46–0.95)		0.78 (0.54–1.14)	
Recessive	T/T‐G/T	1	0.078	1	0.002*	1	0.004	1	0.121
	G/G	0.52 (0.25–1.08)		0.47 (0.29–0.75)		0.44 (0.25–0.76)		0.45 (0.25–0.83)	
Log‐additive	—	0.55 (0.38–0.79)	0.001*	0.82 (0.65–1.02)	0.077	0.65 (0.50–0.85)	0.002*	0.73 (0.55–0.96)	0.027*
rs12722498 (*IL2RA*）									
Dominant	T/T	1	0.292	1	0.772	1	0.622	1	0.865
	T/C‐C/C	1.38 (0.75–2.54)		0.94 (0.67–1.35)		1.11 (0.73–1.69)		0.96 (0.61–1.52)	
Recessive	T/T‐T/C	1	0.999	1	0.474	1	0.924	1	0.999
	C/C	—		0.59 (0.14–2.49)		0.93 (0.20–4.21)		—	
Log‐additive	—	1.23 (0.69–2.17)	0.482	0.93 (0.67–1.29)	0.662	1.08 (0.74–1.59)	0.669	0.89 (0.57–1.38)	0.611
rs228108 (*IL2RB*)									
Domin ant	T/T	1	0.768	1	0.045	1		1	0.438
	T/C‐C/C	0.93 (0.56–1.52)		0.74 (0.55–0.99)		0.55 (0.38–0.78)	0.001	1.16 (0.80–1.67)	
Recessive	T/T‐T/C	1	0.109	1	0.692	1	0.366	1	0.943
	C/C	0.38 (0.12–1.24)		0.89 (0.51–1.57)		0.73 (0.38–1.43)		1.03 (0.15–2.33)	
Log‐additive	—	0.83 (0.56–1.25)	0.388	0.81 (0.64–1.02)	0.078	0.65 (0.49–0.86)	0.003	1.11 (0.82–1.51)	0.502
rs3218264(*IL2RB*)									
Dominant	T/T	1	0.250	1	0.762	1	0.998	1	0.997
	T/C‐C/C	1.38 (0.79–2.47)		0.94 (0.68–1.32)		1.00 (0.67–1.49)		1.00 (0.67–1.50)	
Recessive	T/T‐T/C	1	0.877	1	0.898	1	0.540	1	0.328
	C/C	0.95 (0.53–1.73)		0.97 (0.69–1.37)		0.87 (0.56–1.35)		0.81 (0.52–1.25)	
Log‐additive	—	1.13 (0.78–1.62)	0.521	0.97 (0.79–1.19)	0.791	0.95 (0.73–1.23)	0.716	0.93 (0.72–1.20)	0.568
rs1573673(*IL2RB*)									
Dominant	T/T	1	0.376	1	0.692	1	0.267	1	0.789
	T/C‐C/C	0.80 (0.49–1.31)		0.94 (0.70–1.26)		0.82 (0.57–1.17)		0.95 (0.65–1.39)	
Recessive	T/T‐T/C	1	0.491	1	0.727	1	0.324	1	0.284
	C/C	1.27 (0.64–2.49)		0.92 (0.57–1.48)		1.32 (0.76–2.31)		0.74 (0.42–1.29)	
Log‐additive	—	0.95 (0.67–1.35)	0.789	0.95 (0.76–1.18)	0.642	0.95 (0.73–1.24)	0.718	0.90 (0.98–1.19)	0.462

Abbreviations: 95% CI, 95% confidence interval; OR, odds ratio; RA, rheumatoid arthritis.

*p^a^*‐values were calculated from Wald's test adjusted for age.

*p^b^*‐values were calculated from Wald's test adjusted for gender.

* *p* < 0.05 indicates statistical significance.

### 3.4 Haplotype association

**Figure 1:**
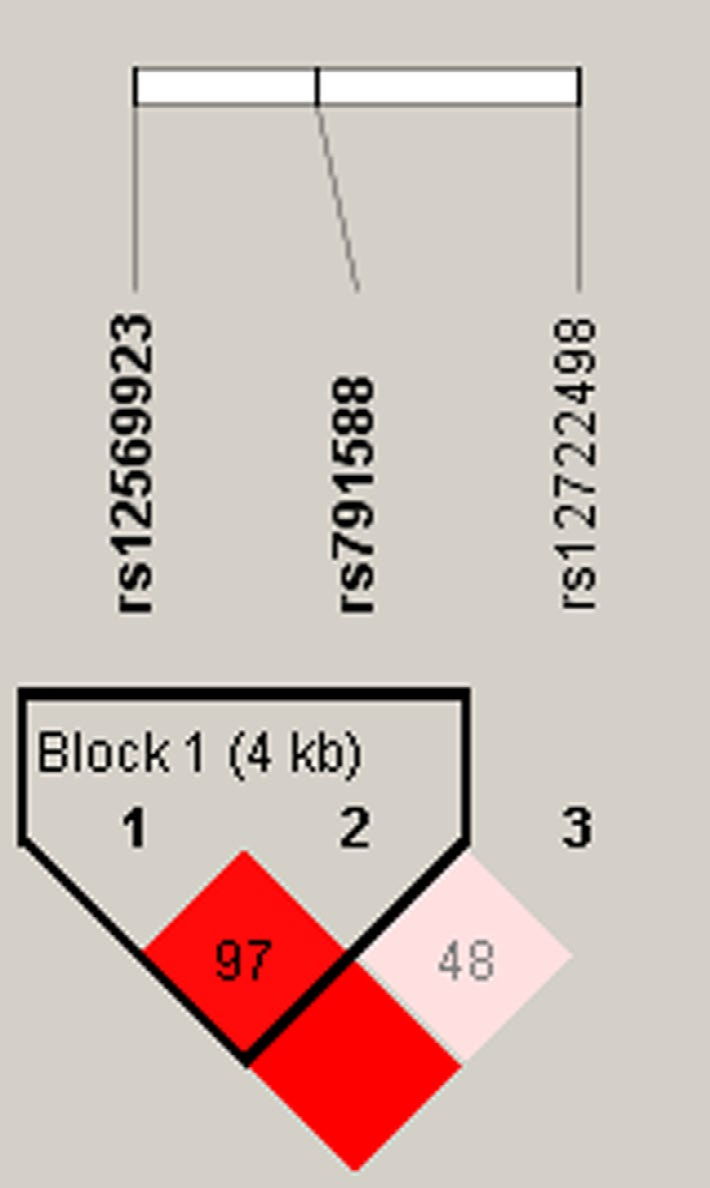
Linkage disequilibrium plots containing three SNPs from *IL1RA *

Finally, allele frequency data from all subjects was used to do the linkage disequilibrium (LD) block, and we found a strong LD in *IL2RA* between rs12569923 and rs791588 (Figure 1). The results of the haplotypes and RA risk are shown in Table 6. There were three haplotypes “C‐G,” “G‐T,” and “C‐T”. However, after unconditional logistic regression analysis adjusted for age and gender, the “C‐G” and “C‐T” haplotypes significantly increased RA risk (for the “C‐G” haplotype: OR = 1.35, 95% CI = 1.12–1.64, *p* = 0.0016; for the “C‐T” haplotype: OR = 1.24, 95% CI = 1.03–1.48, *p* = 0.0016).

**Table 6:** Haplotype analysis results in this study

Block	SNPs	Haplotype	OR (95% CI)	*p* ^a^‐value
Block 1	rs12569923|rs791588	CG	1.35 (1.12–1.64)	0.0016*
		GT	0.89 (0.71–1.12)	0.32
		CT	1.24 (1.03–1.48)	0.021*

Abbreviations: CI, confidence interval; OR, odds ratio; SNP, single‐nucleotide polymorphism

*p* ^a^: Adjusted by gender and age.

* *p* < 0.05 indicates statistical significance.

## 4 DICUSSION

Persistent inflammation and progression of joint damage are the two hallmarks of RA. Several studies showed that the etiology and pathogenesis of RA were likely to comprise a multifactorial disorder resulting from environmental and genetic factors and their interaction. The present case–control study, including 499 RA patients and 507 healthy controls, was aimed to investigate the associations between six SNPs in *IL2RA* and *IL2RB* and the risk of RA in a Chinese Han population. The results showed that rs791588 (*IL2RA*) and rs2281089 (*IL2RB*) may have a protective role against RA risk.

*IL2RA* is located on the short arm of chromosome 10 (10p15‐p14), also known as CD25, which is highly expressed on CD4^+^CD25^+^regulatory T cells (Tregs) and is important for immune homeostasis and the suppression of autoimmune responses (Burchill, Jianying, Vang, & Farrar, 2007). It was initially identified as a candidate gene for T1D and autoimmune disease such as multiple sclerosis. Xia, Qin, and Zhao (2018) found that the loci of *IL2RA* rs2104286 and rs12722489 are closely associated with increasing risk of multiple sclerosis in the Han and Hui nationalities. Abdelrahman et al. (2016) indicated that *IL2RA* was found to be associated with several autoimmune diseases including T1D, and *IL2RA* was more likely to develop T1D (OR = 2.8, *p* = 0.03) in Egyptian children. However, one study showed no significant relationship was observed between the polymorphism of *IL2RA* and T1D in children of northwest of Iran (Ranjouri et al., 2016). Dai et al. (2013) revealed that *IL2RA* was associated with an increased risk of neuromyelitis optica in southern Han Chinese. Another study reported that the variants of *IL2RA* showed a protective role against the intermediate uveitis (Ewald et al., 2015). Based on genetic and serologic study, Knevel et al. (2013) found that inherited altered genetic constitution at *IL2RA* may predispose to a less destructive course of RA. The result of our study showed *IL2RA* (rs791588) was associated with decreasing risk of RA in the Chinese Han population. The difference in results may vary depending on the sample size, ethnicity, test method, and so on.

*IL2RB* encodes the subunit of interleukin‐2 receptor (*IL2R*) and is primarily expressed in the hematopoietic system, where it is involved in the activation of T‐ and NK cell subsets. There is little research on *IL2RB* genes polymorphisms and genetic susceptibility to disease. One study indicated that *IL2RB* polymorphisms do not seem to play a significant role in the non‐anterior uveitis genetic predisposition (Cénit et al., 2013). Another study found that *IL2RB* polymorphisms were not associated with inflammatory bowel disease risk as well. However, Adeline et al. (2014) revealed that a haplotype constructed with two SNPs (rs743777 and rs3218253) located in *IL2RB* was associated with erosive status in early RA. The present study found the polymorphism of *IL2RB* (rs2281089) significantly decreased the risk of RA. The relationship of *IL2RB* gene polymorphisms and other diseases should be investigated in future studies.

Our study aimed to report the association between the polymorphisms of *IL2RA* and *IL2RB* and the risk of RA in the Chinese Han population, which may provide new data to facilitate earlier diagnosis and promote early prevention, and shed light on the new candidate genes and new ideas for the study of subsequent occurrence mechanism of RA. However, some potential limitations of our current study should be considered when decipher the results. This study is only a preliminary basic research; further functional studies and larger population‐based prospective studies are required to understand the genetic factors underlying RA in the subsequent research.

## 5 CONCLUSION

Our results indicate that rs791588 (*IL2RA*) and rs2281089 (*IL2RB*) polymorphisms are associated with RA in a Chinese Han population. These SNPs may serve as prognostic biomarkers for RA in the Chinese Han population.

## CONFLICTS OF INTEREST

The authors have no conflicts of interest to report.
